# Impact of depressive symptoms in outcome of Alzheimer’s disease

**DOI:** 10.1590/S1980-57642010DN40400015

**Published:** 2010

**Authors:** Anita de Paula Eduardo Garavello, Regina Miksian Magaldi, Sérgio Márcio Pacheco Paschoal, Wilson Jacob Filho

**Affiliations:** 1 MD, Geriatric Service, Department of Clinical Medicine, University of São Paulo School of Medicine, São Paulo SP, Brazil.; 2 MD, Assistant Physician, Memory and Aging Unit, Geriatric Service, Department of Clinical Medicine, University of São Paulo School of Medicine, São Paulo SP, Brazil.; 3 MD, PhD, Assistant Physician, Geriatric Service, Department of Clinical Medicine, University of São Paulo School of Medicine, São Paulo SP, Brazil.; 4 MD, Full Professor of the Geriatrics Discipline of the University of São Paulo School of Medicine, São Paulo SP, Brazil.

**Keywords:** Alzheimer’s disease, depression, cognition, caregiver

## Abstract

There is no consensus in the medical literature about the impact of depressive symptoms on the evolution of Alzheimer’s disease (AD).

**Objective:**

To compare the evolution of AD patients, with and without depressive symptoms, in terms of cognition, functionality and caregiver stress.

**Methods:**

The study entailed 2 stages: an initial retrospective stage involving review of medical charts of patients with mild and moderate AD. Patients were divided according to the presence or absence of depressive symptoms, defined by medical interview and questions on depressed mood from the CAMDEX (Cambridge Examination for Mental Disorders of the Elderly) and Neuropsychiatric Inventory (NPI). Twenty-nine patients were evaluated, 37.9% with depression (Group D+) and 62.1% without depression (Group D–). The groups were compared regarding demographic and medical characteristics, cognitive and functional performance, presence of apathy as a separate symptom, and caregiver stress, using standardized tests and questionnaires. In the second transversal step, the same tools were reapplied after 2 to 4 years of follow-up, and evolution for the two groups was compared.

**Results:**

The two groups were highly homogeneous in demographic and clinic characteristics, as well as in length of follow-up, and presented no significant difference in cognitive or functional evaluation at the time of diagnoses or after follow-up. Only caregiver stress was greater in Group D+ at the two time points (p<0.001).

**Conclusions:**

No differences in the evolution of AD patients with or without depressive symptoms were evident. Nevertheless, these symptoms were associated to emotional burden of caregivers.

## Abstract (pt)

Não há consenso na literatura sobre o impacto dos sintomas depressivos na evolução da doença de Alzheimer (DA).

**Objetivo:**

Comparar a evolução de pacientes com DA, com e sem sintomas depressivos, quanto à cognição, à funcionalidade e estresse do cuidador.

**Métodos:**

O estudo incluiu duas etapas: a primeira retrospectiva, com revisão de prontuários de pacientes com DA leve e moderada, divididos conforme presença ou ausência de sintomas depressivos, definidas por anamnese e questões referentes a humor deprimido do CAMDEX (Cambridge Examination for Mental Disorders of the Elderly) e Interrogatório Neuro-psiquiátrico (INP). Foram avaliados 29 pacientes, 37,9% com (D+) e 62,1% sem depressão (D–). Os grupos foram comparados quanto a variáveis demográficas, clínicas e quanto ao desempenho cognitivo, funcionalidade, presença de apatia do paciente e estresse do cuidador, através de questionários e testes padronizados. Na segunda etapa, transversal, foram reaplicados os mesmos instrumentos após 2 a 4 anos de acompanhamento, comparando-se a evolução nos dois grupos.

**Resultados:**

Os grupos, homogêneos segundo variáveis demográficas, clínicas e tempo de evolução, não apresentaram diferença significante nos testes cognitivos e na avaliação funcional, no momento do diagnóstico, e ao longo da evolução. Quanto ao estresse do cuidador, foi maior no grupo D+ nos dois momentos (p<0,001).

**Conclusões:**

Não se constatou diferença na evolução de pacientes com DA com ou sem sintomas depressivos. No entanto, tais sintomas se relacionaram a maior sobrecarga emocional do cuidador, no momento do diagnóstico e na evolução.

Depression and dementia are both prevalent and frequently associated conditions in geriatric patients.^1^ Because they share common symptoms, such as psychomotor slowing, apathy, insomnia and loss of interest, differential diagnoses often becomes a challenge to the physician. Depressive syndrome is present in 30% to 50% of demented patients, especially those with Alzheimer’s disease (AD).^2^

Neuropathology features of AD seem to play a role in depression development, since there is selective loss of noradrenergic cells in *locus ceruleus* and in the serotonergic dorsal raphe nucleus.^3-7^ With this anatomical substrate and the high prevalence of depression in AD, the hypothesis that this mood disorder could be an epiphenomena of this dementia has been raised. However, some studies have shown there are some affective and constitutional symptoms characteristic of mood disorders present only in a subgroup of patients with AD.^2,8,9^ This group can therefore be divided into AD patients with and without depression.

There are conflicting results on whether depression in AD leads to greater cognitive and functional impairment than in control patients without these symptoms,^10,11^ a situation that could worsen caregiver burden and distress and contribute to early institutionalization.^12^

Our aim in this study was to assess the impact of depressive symptoms on cognition and functionality as well as on caregiver stress, in the follow-up of patients with Alzheimer’s disease.

## Methods

The study was carried-out in two stages. The first stage was retrospective and based on medical records of patients registered and followed at the Center of Cognitive Disorders from *Hospital das Clínicas* - São Paulo University Medical School (CEREDIC-HC/FMUSP). Patients were selected based on the following criteria: 60 years old and over; diagnosis of probable or possible Alzheimer’s disease according to the National Institute of Neurological and Communicative Disorders and Stroke and the Alzheimer Disease and Related Disorders Association (NINCDS-ADRDA),^13^ associated with dementia diagnosis according to the Diagnostic and Statistical Manual of Mental Disorder- Fourth Edition (DSM-IV);^14^ mild or moderate AD, based on scores on the Mini Mental State Examination (MMSE), i.e. MMSE ≥9;^15^ patients that had 2 to 4 years of follow-up from diagnoses to end-point of this study (September 2008 to June 2009); use of proper treatment for AD, i.e. inhibitors of acetylcholinesterase at an adequate and stable dose for at least 3 months. Selected patients were then subdivided based on the presence or otherwise of depressive symptoms at the first clinical assessment in the CEREDIC, defined by:

[1] medical interview;
[2] questions regarding depressed mood from the CAMDEX (Cambridge Examination for Mental Disorders of Elderly),^16,17^ considering a positive response as the presence of 2 out of three items;
[3] questions about depression from the Neuropsychiatric Inventory (NPI).^18^ Positivity of item 1 plus item 2 or 3 was considered for determining the presence of depressive symptoms.

The review of the medical records assessed the following variables: age, sex, schooling level, comorbidities as well as use of antidepressant and antipsychotic medications on admission or prescribed at this time. Patients’ performance at first visit was evaluated, as well as at proceeding diagnoses by the following instruments used routinely at the tertiary center:

[A] CAMCOG, a structured interview from the cognitive section of the Cambridge Examination for Mental Disorders of the Elderly (CAMDEX) that allows the identification of cognitive deficits in different domains (orientation, language, memory, attention, concentration, praxia, perception and abstract thinking). Scores range from 0 to 107. Values lower than 79/80 were considered abnormal.^16,17^
[B] Delayed recall of the Brief Cognitive Screening Battery (BCSB 5 min). This is an instrument that allows a quick assessment of several cognitive capabilities such as perception, naming, incidental and immediate memory, and delayed recall of 10 common objects presented as simple drawings. One of the advantages of this test is that it does not suffer a significant influence from schooling.^19,20^
[C] Mini Mental State Examination complements the cognitive assessment.^21,22^
[D] Pfeffer’s Questionnaire of Functional Activities is applied to the caregiver and contains 10 items investigating the patient’s ability to perform instrumental activities of daily life. Scores range from 0 to 30; the higher the score, the higher the patient dependence, indicating functional compromise at scores of 5 and greater.^23^
[E] Zarit Burden Interview: this is a 22 question interview applied to the caregiver, regarding their feelings for the patient. The score is assigned according to frequency of occurrence of items, and ranges between 0 and 88. Higher scores indicate a higher emotional overburden on caregivers.^24^
[F] The report of apathy on the NPI served to evaluate this symptom as independent from other depressive symptoms. This questionnaire applied to the caregiver, tracks 10 specific neuropsychiatric symptoms related to the patient, presenting a score for each domain, calculated as a product of frequency and severity.

The second stage of the study was a transversal analysis in which the same instruments were reapplied after 2 to 4 years of follow-up. Death and institutionalization were exclusion criteria, as these prevented the reapplication of cognitive and functional tests. Patients and caregivers were invited to participate after full explanation of the study objectives, duration and proposed methods. Patients or their legal representatives and caregivers signed the informed consent before the beginning of the evaluations. The study was approved by the Research Ethics Commission of the Institution.

### Statistical analysis

Statistical analysis was performed using SPSS (Statistical Package for Social Sciences) for Windows version 12.0. All tests were performed considering a bilateral hypothesis and assuming a significance level of α=5%. To verify homogeneity of the groups with and without depressive symptoms at the initial evaluation, several characteristics were assessed including: socio-demographic and clinically relevant characteristics (age, schooling, number of comorbidities and medications, performance on cognitive and functional tests, caregiver stress, presence of depressive symptoms and apathy). Quantitative data were presented as medians (interquartile interval) and qualitative data as frequencies. Normality was assessed by Kolmogorov-Smirnov’s test. Groups were compared using the Mann-Whitney test as data were not normally distributed. Fisher’s exact test was used to verify homogeneity of categorical variables. A non-parametric repeated measure ANOVA was applied to compare the evolution of cognitive and functional impairment and of caregivers’ stress in the two groups.

## Results

From the initial 62 selected patients, 53% were excluded due to death, city transference and caregiver refusal (Table 1). The final sample therefore comprised 29 patients, of whom 37.9% (Group D+) had depressive symptoms and 62.1% (Group D–) had no depressive symptoms. Both groups contained a higher proportion of women (63.6% in D+ and 83.3% in D–). As expected, antidepressants use was higher in Group D+ (81.8%) compared with Group D– (11.1%), p<0.001. None of the patients were taking antipsychotics at the time of the initial assessment. Age, schooling, number of comorbidities and medications, and time to reevaluation did not differ between groups.

**Table 1:** Causes of exclusion.

	Group D+ (29)	Group D- (33)
Clinical decompensation	0	1
Death	6	6
Loss of follow-up	3	2
Refuse - city transference Caregiver Patient	4 1 1	0 3 1
Institutionalization	1	1
Severe AD	3	0
Total evaluated	**10 (37.9%)**	**19 (62.1%)**

D+: group of patients with depressive symptoms. D-: group without depressive symptoms. AD: Alzheimer's disease.

Cognitive and functional performance at the first assessment also did not differ significantly by group, while caregivers’ burden was greater in Group D+ (p= 0.003). Scores obtained on the NPI for depression (NPId) and apathy (NPIa) were also higher in Group D+, with p<0.001and p=0.002, respectively (Table 2).

**Table 2:** Comparison of median, inter-quartile interval and p-value between Groups D+ and D- at baseline.

Variables	Depressive symptoms					p-value*
	D+			D-		
	Median	IIQ		Median	IIQ	
Time	36.7	(24.7-40.6)		30.7	(24.8-37.1)	0.550
Age	79.0	(74.0-84.0)		81.0	(78.0-84.3)	0.296
Schooling	4.0	(1.0-4.0)		4.0	(2.0-7.3)	0.580
Number of comorbidities	6.0	(4.0-8.0)		5.0	(3.0-6.3)	0.438
Number of medications	7.0	(3.0-10.0)		5.5	(4.0-8.3)	0.674
MMSE	18.0	(17.0-22.0)		22.5	(17.0-25.3)	0.204
BCSB5min	1.0	(0.0-3.0)		3.5	(0.0-5.3)	0.122
CAMCOG	57.0	(47.0-71.0)		69.0	(56.5-74.5)	0.191
Pfeffer	13.0	(8.0-18.0)		8.0	(4.0-16.5)	0.161
Zarit	33.0	(16.0-40.0)		10.5	(4.3-22.3)	**0.003**
NPId	3.0	(2.0-8.0)		0.0	(0.0-0.0)	**<0.001**
NPIa	8.0	(4.0-8.0)		0.0	(0.0-0.0)	**0.002**

* Mann-Whitney Test. MMSE: Mini-Mental State Examination; BCSB5min: Brief Cognitive Battery/ number of figures recalled after five minutes; CAMCOG: Cambridge Cognitive Examination; Pfeffer: Pfeffer's Questionnaire of Functional Activities; Zarit: Burden Interview; NPId: Neuropsychiatric Inventory of depressive symptoms; NPIa: Neuropsychiatric Inventory of apathy; D+: group with depressive symptoms; D-: group without depressive symptoms.

**Figure 1:**
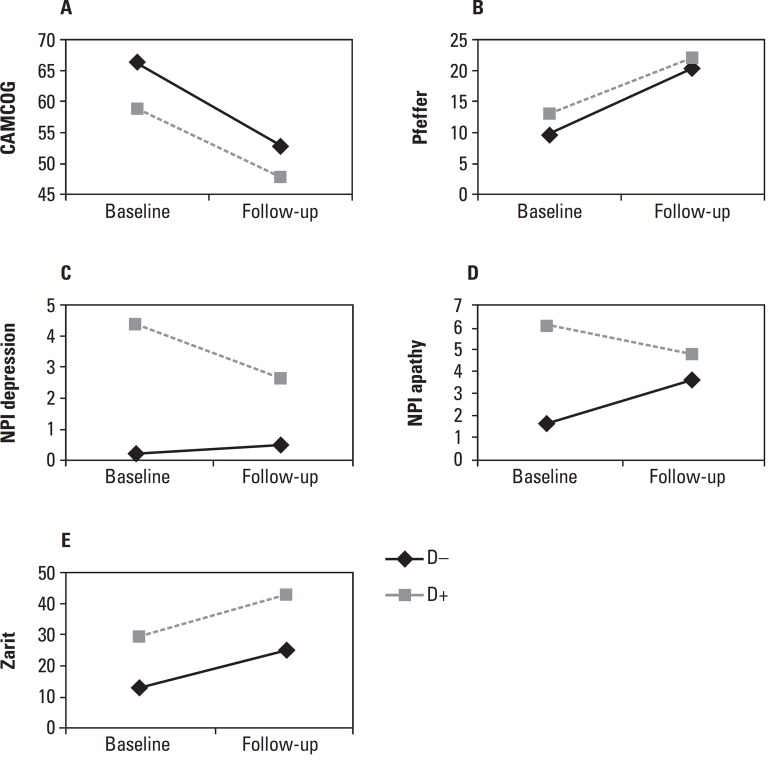
Graph of clinical variable evolution according to time and group. [A] CAMCOG: Cambridge Cognitive Examination. [B] Pfeffer: Questionnaire of Functional Activities. [C] NPId: Neuropsychiatric Inventory of depressive symptoms. [D] NPIa: Neuropsychiatric Inventory of apathy. [E] Zarit: Burden Interview. D+: group with depressive symptoms. D–: group without depressive symptoms. Statistical results in Table 3.

For the CAMCOG cognitive scale, there was a decrease in the scores of both groups, with an intra-individual statistical difference (p=0.031), reflecting the worsening in cognitive abilities with the progression of the disease. However, contrast analysis found a statistically significant decrease only in Group D– (p=0.007) (Figure 1A).

No effect of time, group or interaction was observed on the delayed recall task (BCSB5 min). Functional assessment revealed reduction in both groups over time, evidenced by higher scores on the Pfeffer questionnaire (Figure 1B). Comparison of the two groups detected no between-subject differences in the decrease observed in cognition and functionality (Table 3). In Group D+, a fall in NPId from baseline (p=0.050) was seen, indicating an improvement in depression symptoms throughout the disease course. In Group D– however, no statistically significant difference was observed in NPId score over time (p=0.170), indicating that patients from this group remained free of depressive symptoms. Apathy scores (NPIa) remained stable in Group D+ (p=0.435) but increased in Group D– (p=0.045). As shown in Figure 1E, there was a significant increase in caregiver stress over time in both groups, evidenced by an increase in Zarit’s score, and the level of stress remained higher in Group D+ over the course of the disease (p<0.001).

**Table 3:** Comparison of evolution of groups using non-parametric repeated measures ANOVA.

		Test statistic	g.l..	p-value
BCSB 5min	Between-subjects Within-subjects Interaction	1.56 0.00 1.64	1 1 1	0.211 0.998 0.200
CAMCOG	Between-subjects Within-subjects Interaction	1.00 26.68 1.39	1 1 1	0.315 **<0.001** 0.238
Pfeffer	Between-subjects Within-subjects Interaction	1.52 80.43 0.36	1 1 1	0.217 **<0.001** 0.548
Zarit	Between-subjects Within-subjects Interaction	23.94 21.70 0.10	1 1 1	**<0.001** **<0.001** 0.741
NPId	Between-subjects Within-subjects Interaction	14.16 1.35 5.49	1 1 1	**<0.001** 0.245 0.019
NPIa	Between-subjects Within-subjects Interaction	15.16 0.61 3.73	1 1 1	**<0.001** 0.432 0.053

BCSB5min: Brief Cognitive Battery/ number of figures recalled after five minutes; CAMCOG: Cambridge Cognitive Examination; Pfeffer: Pfeffer's Questionnaire of Functional Activities; Zarit: Burden Interview; NPId: Neuropsychiatric Inventory of depressive symptoms; NPIa: Neuropsychiatric Inventory of apathy; D+: group with depressive symptoms; D-: group without depressive symptoms.

The mean scores, as well as maximum and minimum values, obtained on tests by group are shown in Table 4.

**Table 4:** Scores at baseline and reassessment in Groups D+ and D- on applied questionnaires.

Variables	Depressive symptoms										
	D+						D-				
	N	Minimum	Maximum	Mean	Standard deviation		N	Minimum	Maximum	Mean	Standard deviation
MMSE	11	13	27	19.18	3.92		18	11	26	21.17	4.82
BCSB5min	11	0	4	1.55	1.63		18	0	8	3.17	2.61
CAMCOG	11	34	91	52.77	18.09		17	43	86	66.29	12.75
Pfeffer	11	5	23	13.27	5.69		17	0	20	9.65	6.39
Zarit	11	6	49	29.82	14.78		16	1	39	13.13	10.68
NPId	11	0	12	4.36	3.74		18	0	2	.22	0.64
NPIa	11	0	12	6.09	3.53		18	0	12	1.67	4.01
MMSE2	11	8	24	15.91	5.20		18	5	26	17.22	5.78
BCSB5min2	11	0	5	2.00	1.84		18	0	6	2.67	2.59
CAMCOG2	11	15	82	47.90	20.39		18	18	96	58.00	21.00
Pfeffer2	11	13	30	22.18	5.74		18	6	30	20.44	7.73
Zarit2	10	31	57	43.40	7.67		17	4	49	25.24	12.65
NPI d2	11	0	8	2.64	3.64		18	0	4	.50	1.04
NPIa2	11	0	12	4.82	3.28		17	0	12	3.65	4.27

MMSE: Mini-Mental State Examination; BCSB5min: Brief Cognitive Battery/number of figures recalled after five minutes; CAMCOG: Cambridge Cognitive Examination; Pfeffer: Pfeffer's Questionnaire of Functional Activities; Zarit: Burden Interview; NPId: Neuropsychiatric Inventory of depressive symptoms; NPIa: Neuropsychiatric Inventory of apathy; D+: group with depressive symptoms; D-: group without depressive symptoms; N: number of evaluated patients. 2: on reevaluation, after 2 to 4 years of evolution.

## Discussion

Prevalence of depression in AD is unclear, and depends on the supporting diagnostic criteria employed.^2^ Franch compared different depression diagnosis criteria in AD by applying them to 491 patients, achieving low to moderate levels of concordance. Prevalence ranged from 4.9% on the CID-10 (sub diagnosis) to 43.7% on the NPI.^25^ In the present study, the focus was the presence of depressive symptoms rather than on diagnosing Major Depression, as patients were retrospectively evaluated and no formal diagnostic criteria were applied at first. In a bid to refine this assessment and increase specificity we used 2 different sources of information (CAMDEX and NPI), besides anamneses.

The sample size was a limitation of the present study, in that it decreased the ability to generalize the results. There was significant loss of patients, due to death or city transference, preventing questionnaire reapplication in these cases. Loss of follow-up as well as city relocation (patients were monitored, travelling to each appointment) frequently occurred as a result of substitution of caregivers as the disease advanced, and can be considered an indirect indication of caregiver’s emotional overburden. In Group D+, loss of patients for this reason corresponded to 24% of the sample, contrasting with losses of 6% in the sample for Group D–. Although this is an interesting finding, it does not allow conclusions to be drawn because of the small sample size. Death and institutionalization did not differ significantly between the two groups.

In the group of patients without depressive symptoms, 18.2% were taking antidepressants at baseline. This finding leads us to question whether the depression was over-diagnosed or previously depressed subjects had remission of symptoms with antidepressants, even before accompaniment in our service. Another hypothesis is that antidepressants have been prescribed to treat symptoms such as irritability, insomnia and wandering.

In the initial assessment, patients with and without depressive symptoms were homogeneous in terms of demographic and clinical variables that could influence the evolution of Alzheimer’s Disease such as age, schooling, number of comorbidities and medication, thereby allowing comparison.

There is controversy over whether depression in AD leads to higher functional and cognitive impairment. This relationship was not confirmed in the present study, maybe due to the small sample size. However, previous studies^10,26,27^ such as Holtzer’s study with 536 patients with AD, also failed to find greater cognitive and functional deficit in patients with depressive symptoms. By contrast, De Ronchi and Kales observed greater functional compromise in patients with AD associated with depressive symptoms.^28,29^ However, the former study did not report the depression treatment while the second study stated that only 35% of the patients with depressive symptoms received antidepressants, contrasting with 81.8% in Group D+ of our study, raising the question as to whether antidepressant use may have prevented greater functional loss.^30^

Apathy increase or maintenance and depressive symptoms decrease found after 2 to 4 years in the evolution of AD patients corroborates results from the literature. Holtzer observed a fall in depression prevalence from 40% to 28% after 4 years.^10^ Starkstein accompanied 65 AD and depressive patients, diagnosed according to DSM- IV and Hamilton Rating Scale, and observed remission of 50% in depressive symptoms after 1 year and a half, but persistence of apathy.^11^ This reinforces the notion that depression and apathy are separate domains of dementia,^31^ in spite of being strongly correlated.^26^

Other possible limitations of this study include the time of reassessment of the patients and the study design, whereby data were collected at two separate time points with no evaluation of events during the interim period. Patients were evaluated after 2-4 years of evolution, given that no significant clinical change would be noted within one year. However, during the interim period, patients without depressive symptoms at baseline (Group D–) may have developed these symptoms prior to the second review, precluding the identification and implications of the outcome. Longitudinal studies involving larger samples and with shorter intervals between assessments should be conducted to confirm these findings.

Presence of depression in AD patients in this study was correlated to higher caregiver stress, a finding consistent with results of Hurt’s study, in which irritability and depressive symptoms on the Neuropsychiatric Inventory (NPI) applied in these patients were a predictor of low quality of life of the caregiver.^32^ Schulz demonstrated that the reporting of emotional suffering in dementia patients was significantly associated to depressive symptoms in the caregiver.^33^

In conclusion, depressive symptoms were not associated to worst evolution of cognition and functionality in mild to moderate AD patients. However, caregivers of these patients showed greater emotional burden both at the beginning and throughout the disease when, besides AD, patients also had depressive symptoms. These results highlight the importance of devising strategies for relieving caregiver stress, which tends to mount with disease progression despite the use of adequate treatment for dementia and depression.
